# Structure of the lutein-binding domain of human StARD3 at 1.74 Å resolution and model of a complex with lutein

**DOI:** 10.1107/S2053230X16010694

**Published:** 2016-07-27

**Authors:** Martin P. Horvath, Evan W. George, Quang T. Tran, Kody Baumgardner, Gabe Zharov, Sarah Lee, Hassan Sharifzadeh, Saeed Shihab, Ty Mattinson, Binxing Li, Paul S. Bernstein

**Affiliations:** a Department of Biology, University of Utah, 257 S 1400 E, Salt Lake City, UT 84112, USA; b Department of Ophthalmology and Visual Sciences, Moran Eye Center, University of Utah School of Medicine, Salt Lake City, UT 84132, USA

**Keywords:** carotenoid-binding protein, START domain, StARD3, lutein, protein tunnels and cavities

## Abstract

A crystal structure of the lutein-binding domain of human StARD3 (StAR-related lipid-transfer protein 3; also known as MLN64) has been refined to 1.74 Å resolution. A previous structure of the same protein determined to 2.2 Å resolution highlighted homology with StARD1 and shared cholesterol-binding character. StARD3 has since been recognized as a carotenoid-binding protein in the primate retina, where its biochemical function of binding lutein with specificity appears to be well suited to recruit this photoprotective molecule. The current and previous structures correspond closely to each other (r.m.s.d. of 0.25 Å), especially in terms of the helix-grip fold constructed around a solvent-filled cavity. Regions of interest were defined with alternate conformations in the current higher-resolution structure, including Arg351 found within the cavity and Ω1, a loop of four residues found just outside the cavity entrance. Models of the complex with lutein generated by rigid-body docking indicate that one of the ionone rings must protrude outside the cavity, and this insight has implications for molecular interactions with transport proteins and enzymes that act on lutein. Interestingly, models with the ∊-ionone ring characteristic of lutein pointing towards the bottom of the cavity were associated with fewer steric clashes, suggesting that steric complementarity and ligand asymmetry may play a role in discriminating lutein from the other ocular carotenoids zeaxanthin and *meso*-zeaxanthin, which only have β-ionone rings.

## Abstract 2

The structure of a START-domain protein known to bind lutein in the human retina is reported to an improved resolution limit. Rigid-body docking demonstrates that at least a portion of lutein must protrude from the large tunnel-like cavity characteristic of this helix-grip protein and suggests a mechanism for lutein binding specificity.

## 1. Introduction   

The macula lutea (yellow spot) at the center of the primate retina is enriched in the xanthophyll carotenoids lutein, zeaxanthin and *meso*-zeaxanthin. These carotenoids are thought to protect the human eye from photo-oxidative stress (Bernstein *et al.*, 2016 ▸; Li *et al.*, 2010 ▸; Beatty *et al.*, 1999 ▸). Indeed, epidemiological studies and prospective clinical trials have shown that dietary intake and supplementation with lutein and zeaxanthin increase the likelihood of avoiding age-related macular degeneration (AMD), a leading cause of blindness (Seddon *et al.*, 1994 ▸; Age-Related Eye Disease Study 2 Research Group, 2014 ▸; Wu *et al.*, 2015 ▸; Bernstein *et al.*, 2016 ▸). The majority of these carotenoid molecules are specifically localized in the outer plexiform layers (also known as the Henle fiber layer) of the human fovea. GSTP1 and StARD3 (also known as MLN64) have been identified as the zeaxanthin-binding and lutein-binding protein in the human macula, respectively, and these are thought to be responsible for the specific tissue distribution and stability of the ocular carotenoids (Bhosale *et al.*, 2004 ▸; Li *et al.*, 2011 ▸). StARD3 was initially identified as a lutein-binding protein because of its high degree of homology to the carotenoid-binding protein found in silkworm (Li *et al.*, 2011 ▸). Its retina-related role was confirmed through the examination of tissue-specific expression patterns, and binding studies monitored by surface plasmon resonance (SPR) demonstrated that StARD3 binds lutein with affinity and specificity (Li *et al.*, 2011 ▸). The lutein-binding function of StARD3 resides within the C-terminal START domain comprising residues 216–444, hereafter referred to as StARD3_LBD_ (Li *et al.*, 2011 ▸).

Interest in StARD3 predates the discovery of its lutein-binding function and retina-protective role. Its X-ray crystal structure was determined by the Hurley laboratory as a surrogate for StARD1 (Tsujishita & Hurley, 2000 ▸). StARD1 mobilizes cholesterol as the first committed step in steroid biogenesis, and homology between StARD3 and StARD1 suggested that StARD3 also binds cholesterol (Watari *et al.*, 1997 ▸; Tsujishita & Hurley, 2000 ▸). The most remarkable structural feature characteristic of StARD3 and StARD1 is a large (∼1000 Å^3^) solvent-filled cavity (Tsujishita & Hurley, 2000 ▸; Thorsell *et al.*, 2011 ▸).

The structural basis by which StARD3 and GSTP1 recognize, bind and protect lutein, zeaxanthin and *meso*-zeaxanthin is not known. With the overarching goal of providing a molecular picture for carotenoid recruitment to the human retina, we have targeted carotenoid complexes of StARD3_LBD_ and GSTP1 for structure determination. Structures of these proteins in their carotenoid-complexed forms have been elusive because conditions which solubilize the hydrophobic lutein and zeaxanthin molecules require either detergent or organic solvents, and these agents have been determined to prevent crystal growth and to damage preformed crystals.

While these challenges are being resolved, we have obtained high-resolution X-ray diffraction data for the un­liganded proteins and report here the structure of StARD3_LBD_ refined to 1.74 Å resolution. This structure corresponds closely to the original 2.2 Å resolution structure (Tsujishita & Hurley, 2000 ▸). The new structure includes additional solvent molecules and alternate conformations for several residues, including Arg351, which is located within the cavity, and residues Gly336–Gly339, which are part of the omega loop found just outside the cavity entrance. Model-building experiments indicate that at least one of the ionone rings of lutein must protrude through a cavity entrance. The current structure and its lutein-complexed model are discussed in relation to mechanisms for carotenoid binding specificity and the molecular interactions necessary for the transport, protection and metabolism of carotenoids in the human eye.

## 2. Methods   

### 2.1. Cloning of an expression system for tagless StARD3_LBD_   

The tagless StARD3_LBD_-pET-22b(+) expression system was obtained by ligation-independent cloning (Aslanidis & de Jong, 1990 ▸; Li & Elledge, 2007 ▸). Briefly, DNA encoding residues 216–444 of human StARD3 was amplified by PCR using a His_6_-tagged StARD3 expression plasmid as the template. Primers for this PCR reaction were designed to avoid the His_6_ tag and to include ∼20 bp of DNA flanking the NdeI/EcoRI insertion site present in pET-22b(+). This StARD3-encoding DNA was assembled with the pET-22b(+) vector by mixing with two DNA fragments obtained by amplifying the following regions from pET-22b(+): 287–3268 (contains an NdeI site and the *lacI* gene) and 3245–197 (contains an EcoRI site and the *bla* gene). Each of the three PCR products was purified by electrophoresis in a 0.85% agarose gel prior to mixing and transformation directly into heat-shock competent DH5α *Escherichia coli* cells. Ampicillin-resistant colonies were screened by restriction-enzyme digestion and DNA sequencing of miniprep DNA. The resulting T7 promoter-driven expression vector encodes residues 216–444 of StARD3, as confirmed by mass spectrometry.

### 2.2. Purification of tagless StARD3_LBD_   

The lutein-binding domain of StARD3 was expressed in *E. coli* Origami B (DE3) cells (Novagen) and purified by ammonium sulfate fractionation followed by ion-exchange and size-exclusion chromatography. The concentration of StARD3_LBD_ was determined from the absorbance at 280 nm using an extinction coefficient of 1.1 ml mg^−1^ cm^−1^ (0.028 µ*M* ^−1^ cm^−1^) calculated by the method of Gill & von Hippel (1989 ▸).

#### 2.2.1. Growth of bacterial cultures   

Bacterial cultures were cultivated with shaking in 2×YT medium supplemented with 30 m*M* potassium phosphate pH 7.5, 5 m*M* glucose, 10 µg ml^−1^ kanamycin, 12.5 µg ml^−1^ tetracycline and 500 µg ml^−1^ ampicillin. A 750 ml culture was expanded at 310 K. At an OD_600 nm_ of 0.7–0.9, the culture was chilled on ice before adding IPTG to a final concentration of 100 µ*M*. Induction proceeded at 290 K with shaking for 24–30 h prior to harvesting the cells by centrifugation at 4000*g*. The cells were washed in 10 m*M* Tris, 1 m*M* EDTA pH 8.2, 0.5 m*M* phenylmethylsulfonyl fluoride (PMSF) and collected by centrifugation prior to freezing at 253 K.

#### 2.2.2. Ammonium sulfate precipitation   

Frozen cells were submerged in 100 g lysis buffer consisting of 0.1 *M* sodium chloride, 0.05 *M* Tris pH 8, 1 m*M* EDTA. Upon thawing, cells were lysed by sonication on ice. The lysate was centrifuged at 20 000*g*, and the supernatant was collected in an ice-cold glass beaker. Solid ammonium sulfate was added slowly with stirring on ice to achieve 30% saturation (17.5 g ammonium sulfate per 100 g supernatant). After 30 min, the sample was centrifuged at 15 000*g* for 20 min. Additional ammonium sulfate was added slowly with stirring on ice to achieve 65% saturation (an additional 22.5 g ammonium sulfate per 100 g supernatant). After 40 min, the second ammonium sulfate fraction was collected by centrifugation at 15 000*g* for 15 min. The pellet was dissolved by adding 20 ml of freshly prepared, ice-cold dialysis buffer consisting of 50 m*M* sodium chloride, 25 m*M* HEPES pH 6.5, 0.25 m*M* EDTA, 1 m*M* dithiothreitol (DTT). The sample was dialyzed against 1.5 l dialysis buffer at 277 K without stirring overnight.

#### 2.2.3. Ion-exchange and size-exclusion chromatography   

**Figure 1:**
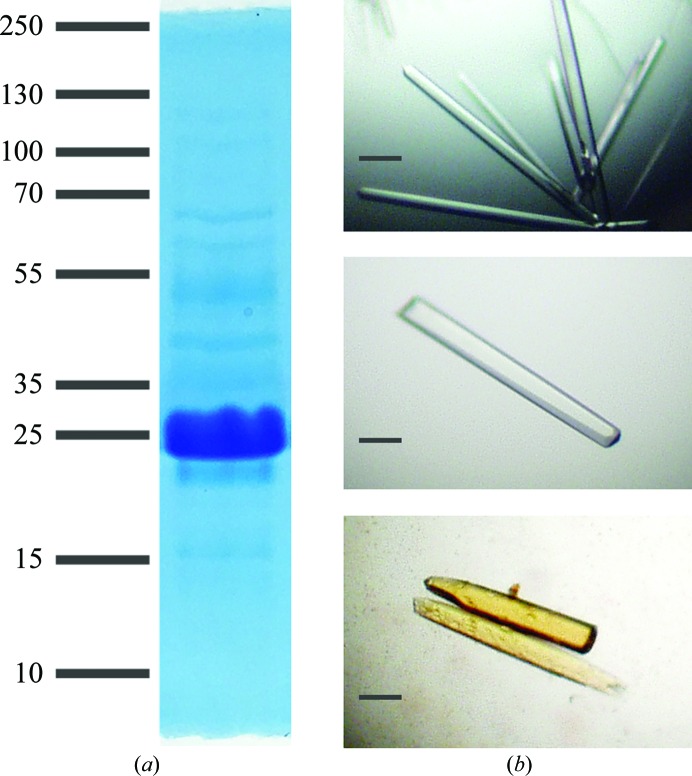
Purity and crystals of StARD3_LBD_. (*a*) Purified StARD3_LBD_ (15 µg) was analyzed by SDS–PAGE and stained with Coomassie Blue. The most prominent (∼90%) protein co-elutes with a 25 kDa molecular-weight marker (expected mass 29 953 Da). (*b*) Rod-shaped crystals grew in 1–2 d. The scale bar indicates 100 µm. Crystals of StARD3_LBD_ are colorless (top, middle) and acquire a golden color when stored in solutions also containing powdered lutein (bottom).

The dialysate was centrifuged at 20 000*g* in an ice-cold rotor for 20 min, filtered through a low-protein-binding 0.45 µm filter and loaded by gravity onto two 2 ml SP Sepharose columns in parallel, each equilibrated in dialysis buffer. The average flow rate was approximately 1 ml min^−1^. Columns were washed with 10 ml dialysis buffer and eluted by increasing the concentration of sodium chloride stepwise with 5 ml volume per step. Fractions enriched in StARD3_LBD_ were identified by SDS–PAGE stained with Coomassie, pooled and concentrated to a volume of 0.3 ml before filtering using a 0.45 µm Nanosep spin filter (Pall Life Sciences) and injecting onto a size-exclusion column equilibrated with SEC buffer consisting of 20 m*M* Tris pH 7.5, 150 m*M* sodium chloride, 2 m*M* DTT. Fractions enriched in StARD3_LBD_ protein were pooled and concentrated to 8 mg ml^−1^ (Fig. 1 ▸ *a*).

### 2.3. Crystallography   

#### 2.3.1. Crystallization   

Crystals of StARD3_LBD_ were obtained by the hanging-drop vapor-diffusion method as described previously (Tsujishita & Hurley, 2000 ▸). Well solutions consisting of 0.1 *M* CHES pH 8.6–9.4, 0.2 *M* lithium sulfate, 0.8–0.96 *M* sodium/potassium tartrate, 0.01 *M* DTT were combined with equal volumes (1.5–3 µl) of protein solution. Rod-shaped crystals appeared within 2 d and grew to a maximum size within one week at room temperature (Fig. 1 ▸ *b*). Crystals were moved to matched solutions additionally containing 15–20% ethylene glycol prior to cooling in liquid propane and storage under liquid nitrogen.

#### 2.3.2. Data collection   

Crystals were measured in oscillation mode on the SIBYLS beamline 12.3.1 at the Advanced Light Source (Classen *et al.*, 2013 ▸) using synchrotron radiation tuned to 1.116 Å. The rod morphology made it possible to reposition the crystal periodically during the course of data collection so as to obtain complete and redundant data without overexposing any one portion. Indexing and strategy development was accomplished with *HKL*-2000 (Otwinowski & Minor, 1997 ▸). Complete data sets were processed with *XDS* and *XSCALE* (Kabsch, 2010*a* ▸,*b* ▸).

#### 2.3.3. Refinement   

Phases were obtained by placing PDB entry 1em2 (Tsujishita & Hurley, 2000 ▸) into the nearly isomorphous unit cell by rigid-body refinement. Refinement continued with several iterative cycles of positional and temperature-factor adjustment with *PHENIX* and model adjustment with *Coot* (Adams *et al.*, 2010 ▸; Emsley *et al.*, 2010 ▸). Early refinement rounds additionally applied torsion-angle simulated annealing with a temperature protocol descending from 1500 K to a final temperature of 50 K.

### 2.4. Model construction of the complex with lutein   

Structural models of protein-complexed lutein were obtained from the light-harvesting complexes of spinach and pea with the following PDB entries: 1rwt (Liu *et al.*, 2004 ▸), 3pl9 (Pan *et al.*, 2011 ▸), 4xk8 (Qin *et al.*, 2015 ▸), 4y28 (Mazor *et al.*, 2015 ▸) and 3jcu (Wei *et al.*, 2016 ▸). Selected lutein molecules were manually positioned within the StARD3 structure to generate a number of docked templates. An ensemble of 27 324 structures was constructed by superposition of the 40 experimentally determined lutein structures from PDB entries 1rwt, 3pl9, 4xk8 and 3jcu onto these docked template molecules with variations of least-squares alignments either directly or with inverting molecule orientation. Ensemble members were scored by determining the number of clashes and the number of potential hydrogen bonds with use of a C language program written by MPH. Frequency analysis to detect the orientation preference was limited to the 8469 ensemble members that belonged to the ‘one portal’ set and that scored at least one potential hydrogen bond.

## 3. Results   

### 3.1. Protein expression and crystallization   

Initial attempts to prepare crystals of StARD3_LBD_, purified as described previously (Tsujishita & Hurley, 2000 ▸), yielded small crystals with poor diffraction quality. We therefore cloned a T7 promoter-driven expression system that produces StARD3_LBD_ without a tag, with the idea that the challenge in producing crystals may have related to the removal of the His_6_ tag by protease treatment. Purification by means of ammonium sulfate fractionation and two chromatography steps (and no proteolysis treatment) yielded 5 mg protein from 0.75 l bacterial culture (Fig. 1 ▸ *a*). The yield and purity were lower than that obtained with the tagged version; however, the resulting protein readily produced large (50 × 50 × 500 µm) rod-shaped crystals (Fig. 1 ▸ *b*) which diffracted synchrotron radiation to 1.7 Å resolution (Table 1 ▸).

### 3.2. Structure of StARD3_LBD_   

#### 3.2.1. Structure determination   

The structure of StARD3_LBD_ was determined by refinement against the newly measured 1.74 Å resolution data, starting with rigid-body placement of a previously determined structure (Tsujishita & Hurley, 2000 ▸). The starting model (PDB entry 1em2) is 99% identical to the wild-type StARD3_LBD_ determined in the current work, and both proteins crystallized in the same *P*3_1_21 space group with highly comparable unit-cell parameters. The higher resolution limit reported here probably reflects differences in crystal size (the present crystals are twice as large in each dimension), differences in synchrotron source flux (ALS beamline 12.3.1 for the present study *versus* NSLS beamline X4A for the previous study) and improvements in detector capabilities (ADSC Q315r *versus* ADSC Q4). The final structural model includes residues 231–444 of StARD3 (the coordinates for residues 216–230 were omitted because no electron density was observed), 224 water molecules, three molecules of ethylene glycol, one sulfate ion and one molecule of l-(+)-tartaric acid. The *R*_work_ and *R*_free_ values are 0.169 and 0.192, respectively. Table 2 ▸ reports statistical measures for model validation.

#### 3.2.2. Structure overview   

**Figure 2:**
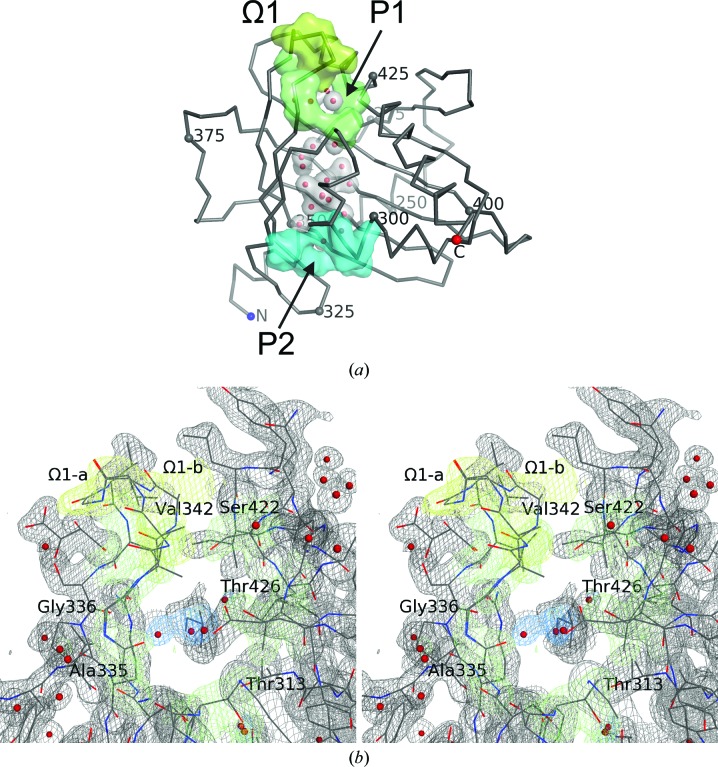
Structure overview and representative electron density for StARD3_LBD_. (*a*) The C^α^ trace of StARD3 is shown along with surfaces for key structural features: the omega loop (Ω1, lemon), portal 1 (P1, lime), portal 2 (P2, cyan) and solvent molecules located inside the cavity (red spheres inside gray molecular surfaces). (*b*) A stereoview of electron density and protein structure at portal 1 is shown. Electron density was calculated as a simulated-annealing composite OMIT map with 2|*F*_o_| − |*F*_c_| coefficients and is contoured at 1.1σ. Map color indicates residues belonging to the omega loop (lemon), portal 1 (lime) and tunnel solvent (blue). Both conformations of the omega loop are shown (Ω1-a and Ω1-b). Residues lining portal 1 are labeled (Thr313, Ala335, Gly336, Val342, Ser422 and Thr426).

Fig. 2 ▸ shows an overview of the protein as well as representative electron density. As described previously (Tsujishita & Hurley, 2000 ▸), StARD3_LBD_ adopts the helix-grip fold with a nine-stranded curved β-sheet and three α-helices coalescing around a large solvent-filled cavity. In helix-grip proteins the cavity entrance is guarded by an omega loop (Ω1) that connects two β-strands (β5 and β6 in StARD3_LBD_). The current structure is highly correlated with the 2.2 Å resolution structure (Tsujishita & Hurley, 2000 ▸). Superposition yields a root-mean-square deviation (r.m.s.d.) of 0.25 Å for 210 C^α^ atoms. The main differences in the structure involve the restoration of the wild-type residues Met307, Phe388 and Met427 (these residues were substituted with selenomethionine in PDB entry 1em2) and the modeling of 17 residues with alternate conformations, including Arg351, which is inside the cavity, and residues Gly336–Gly339, which are part of Ω1 near the cavity entrance.

#### 3.2.3. Tunnel-like cavity   

A striking feature of StARD3_LBD_ is the prominent tunnel-like cavity lined by hydrophobic and polar residues with sufficient volume to accommodate a molecule of cholesterol, as demonstrated by modeling (Tsujishita & Hurley, 2000 ▸). The tunnel-like cavity measures ∼20 Å long from end to end, curves slightly and accommodates 18 solvent molecules, one of which is an ethylene glycol in the current structure (Fig. 2 ▸). Electron-density maps did not reveal any larger molecules located inside the cavity. The cavity communicates with bulk solvent through two openings, which we will call portal 1 and portal 2 (Fig. 2 ▸). Portal 1 is wider in diameter compared with portal 2 (4  × 8 Å for portal 1 *versus* 2 × 4 Å for portal 2; distances are measured between solvent-accessible surfaces). Indeed, structural descriptions of helix-grip proteins generally speak of only one cavity entrance.

#### 3.2.4. Alternate conformations   

**Figure 3:**
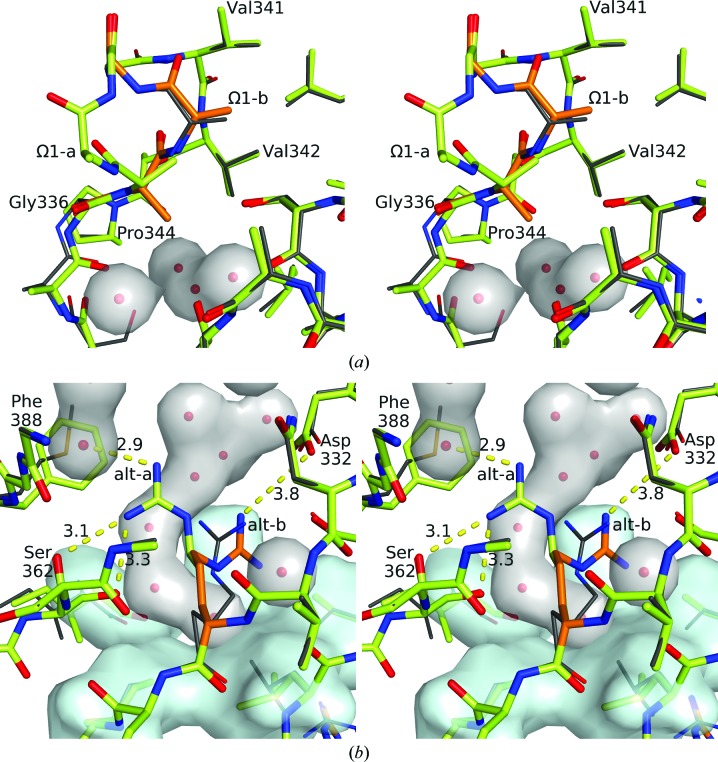
Alternate conformations for the omega loop (*a*) and Arg351 (*b*) as stereoviews. The structure of StARD3_LBD_ reported here is shown superimposed with the previously determined structure (PDB entry 1em2; charcoal C atoms). Protein residues, including the A conformation, are shown with C atoms colored lime. The B conformation is shown with C atoms colored orange. Solvent molecules located in the tunnel-like cavity are shown as red spheres inside gray molecular surfaces. (*a*) Ala338 experiences the largest displacement (6.6 Å for C^β^), moving away from the cavity entrance upon switching from the Ω1-b to the Ω1-a conformation. (*b*) Hydrogen bonds and salt bridges evident for Arg351 in each of two alternate conformations are indicated with dashed yellow lines and distance measurements (Å). In this panel, portal 2, shown as a molecular surface, comprises residues Pro304, Ile319, Leu328 and Ile353, as well as the main-chain atoms of residues 301–303. Residue 388 is Phe (wild type) in the current structure and selenomethionine in the previously reported structure. For clarity, some residues and solvent molecules are not shown.

Structural flexibility is evident for residues located near the cavity portals. A segment of four residues (Gly336, Ala337, Ala338 and Gly339) was associated with weaker-than-average electron density along two alternative chain paths, suggesting that the Ω1 loop is capable of movement just outside portal 1. These residues were modeled in two alternative conformations (Fig. 3 ▸ *a*), one of which was seen in the original structure (Ω1-b; occupancy = 0.44). The other conformation (Ω1-a; occupancy = 0.56) differs principally by a flip in the peptide bond connecting Ala338 and Gly339, which results in a maximal 6.6 Å displacement away from the tunnel opening experienced by C^β^ of residue Ala338 (Figs. 2 ▸ *b* and 3 ▸ *a*). Smaller displacements are observed for the neighboring residue Ala337, which rotates by approximately 90°. In addition to the alternate conformations observed outside portal 1, the side chain of Arg351 was resolved as two alternative conformations inside the tunnel close to portal 2 (Fig. 3 ▸ *b*). One of these reaches across the cavity to form a salt bridge with Asp332 and is most similar to the conformation of this residue reported previously (Arg351-b; occupancy = 0.43). The second conformation (Arg351-a; occupancy = 0.57) adopts a different rotamer and forms hydrogen bonds to solvent molecules and the carbonyl and hydroxyl O atoms of Ser362. Structural flexibility in these regions may be necessary for the binding of bulky ligands such as lutein. Alternate conformations are also evident for residues 276–280, which are located on the surface of StARD3_LBD_ and do not directly impact tunnel accessibility.

### 3.3. Building a model of the complex with lutein   

#### 3.3.1. Lutein structure   

**Figure 4:**
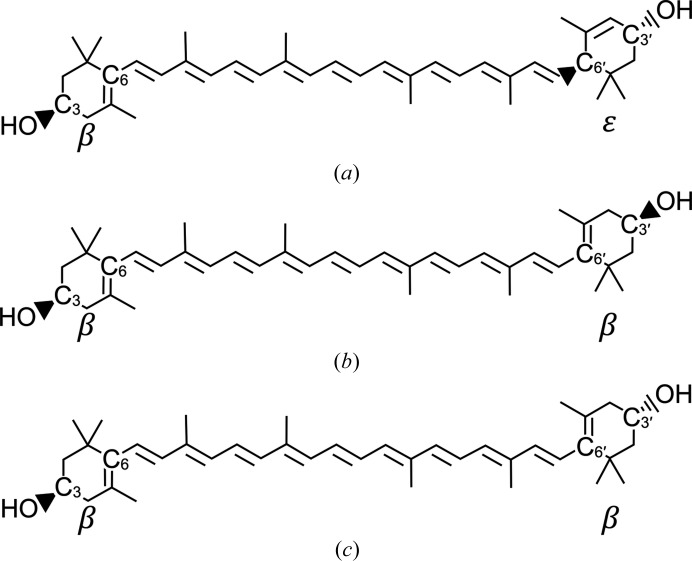
Chemical structures of lutein (*a*), zeaxanthin (*b*) and *meso*-zeaxanthin (*c*). The ionone rings of lutein have different shapes. The β-ring contains a single stereocenter (3*R*); the ∊-ring contains two stereocenters (3′*R*, 6′*R*). Asymmetry in the ionone shape distinguishes lutein from the other ocular xanthophylls and may contribute to selectivity for lutein and exclusion of zeaxanthin and *meso*-zeaxanthin, which each have two β-ionone rings (3*R* and 3′*R* in zeaxanthin; 3*R* and 3′*S* in *meso*-zeaxanthin).

Lutein (C_40_H_56_O_2_) belongs to the xanthophyll carotenoids synthesized by plants and bacteria. Two ionone rings, each bearing a hydroxyl group, are connected by a long polyene backbone. The molecular structure includes three defined stereocenters (Fig. 4 ▸). We examined the structures of protein-complexed lutein molecules found as part of the light-harvesting complexes from spinach and pea (Liu *et al.*, 2004 ▸; Pan *et al.*, 2011 ▸; Qin *et al.*, 2015 ▸; Wei *et al.*, 2016 ▸). All examples show curvature in the polyene backbone, steric complementarity with surfaces of the proteins, especially at ionone ring-contacting pockets, and two or three hydrogen bonds formed between each of the hydroxyl groups of lutein and acceptor and donor groups presented by the chlorophyll-binding protein subunits. The average length measured between hydroxyl O atoms is 30.5 ± 0.2 Å. The size and shape of lutein are thus closely matched to the size and shape of the cavity found in StARD3_LBD_, but it would be impossible to completely fit the entire molecule into the cavity. Interestingly, crevices and protrusions of the protein found just outside each portal appear to be articulated to specifically accommodate some type of ligand. We propose that structural features close to and outside one or both of the portals make contact with an ionone ring, similar to the ionone ring-binding pockets observed in light-harvesting complex structures (Liu *et al.*, 2004 ▸; Pan *et al.*, 2011 ▸; Qin *et al.*, 2015 ▸; Mazor *et al.*, 2015 ▸; Wei *et al.*, 2016 ▸).

#### 3.3.2. Rigid-body docking   

**Figure 5:**
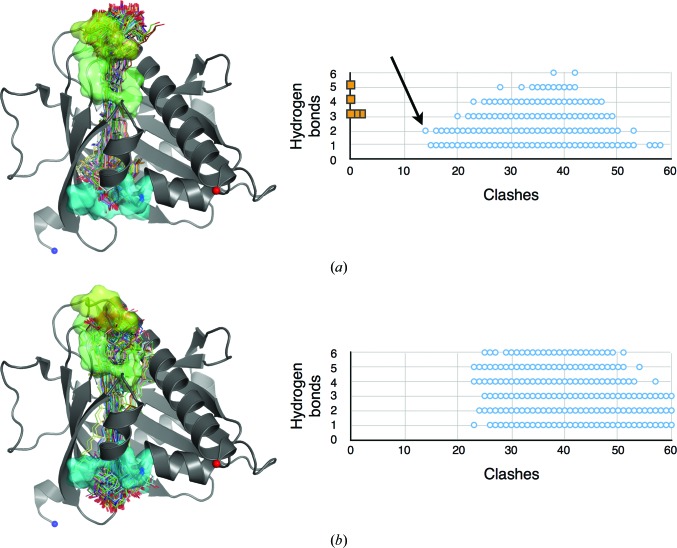
Lutein molecules docked with StARD3. The ensemble can be divided into two sets shown in (*a*) and (*b*). (*a*) Members of this set protrude only through portal 1 (*n* = 13 068; ‘one portal’). (*b*) Members of the other set breach both portals (*n* = 14 256; ‘two portals’). Clashes and potential hydrogen bonds are plotted for members of each set that scored at least one but not more than six hydrogen bonds (blue open circles). For reference, scores measured for experimentally determined structures of protein-complexed lutein molecules are also plotted [gold-filled squares in (*a*)]. The best scoring molecules from the ‘two portals’ set incurred 23 clashes. The overall ‘winner’ belonged to the ‘one portal’ set shown in (*a*), incurred 14 clashes and realised two potential hydrogen bonds (an arrow points to the data point characterizing this winner).

To test this idea, lutein molecules were docked within the StARD3_LBD_ structure (Fig. 5 ▸). Several different lutein molecules were tested, each derived from an experimental structure of a light-harvesting complex. The omega loop was assumed to be in its Ω1-a conformation, and Arg351 was likewise restricted to the conformation that hydrogen bonds to Ser362 (not salt-bridged with Asp332) because these conformations appeared to be most accommodating with respect to avoiding steric clashes. Except for translation and rotation, no structural adjustments were permitted in either the lutein ligand or the StARD3_LBD_ protein. Each docking outcome was scored for clashes (un­favorable, closer than 3 Å contact) and hydrogen bonds (favorable, 2.0–4.2 Å distance from hydroxyl O atom to donor or acceptor). We realise that these distance cutoffs are generous by comparison with strict stereochemical rules, but judge them to be reasonable when considering that adjustments in structure are to be anticipated. The ‘winner’ incurred fewer steric clashes than any of the other outcomes and realised two hydrogen bonds, one for each of the two hydroxyl groups of lutein (Fig. 5 ▸).

#### 3.3.3. Orientation preference   

**Figure 6:**
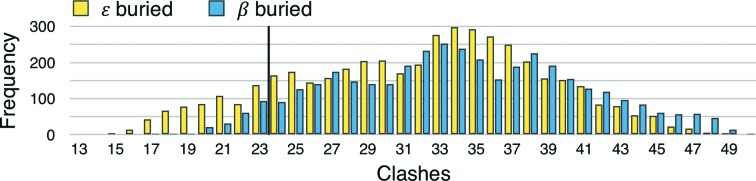
Orientation preference for lutein. The frequency of observing docked lutein–StARD3_LBD_ complexes is plotted as a function of steric clashes incurred for members of the ‘one portal’ set with at least one potential hydrogen bond. Within the top 10% scoring group (fewer than 23 clashes; 836 of 8469 outcomes), the frequency for lutein molecules oriented with the ∊-ionone ring buried deep inside the cavity (yellow bars; *n* = 620) was 2.9-fold higher compared with the frequency for the opposite orientation with the β-ionone ring buried (blue bars; *n* = 216). This frequency difference meets the criteria for statistical significance with *p* < 0.00001 for a chi-squared test. Similarly strong frequency differences indicating that it is easier to place the ∊-ionone ring inside the tunnel-like cavity and the β-ionone ring outside portal 1 were observed if the analysis was repeated with Ω1 and Arg351 adopting their alternate conformations or with Ω1 deleted and Arg351 truncated to an alanine residue.

**Figure 7:**
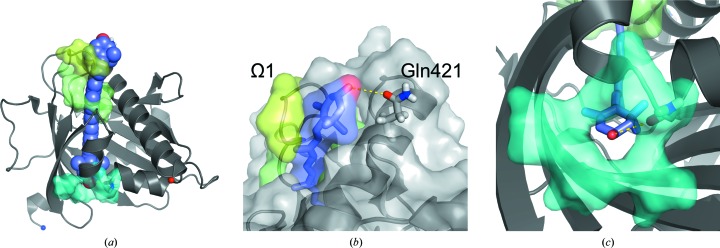
‘Winner’ lutein molecule docked with StARD3. (*a*) Overview of the StARD3_LBD_ structure shows the best-scoring lutein candidate from the ‘one portal’ set. (*b*) Steric complementarity and a potential hydrogen bond to Gln421 are apparent outside portal 1 for the overall ‘winner’ lutein molecule. (*c*) The ‘winner’ lutein molecule positions the hydroxyl group of the ∊-ionone ring just inside portal 2, where it finds a potential hydrogen-bonding partner with the peptide bond connecting residues Ile301 and Leu302.

The ensemble of 27 324 docked lutein molecules included four overlapping sets: a set that penetrated both portals (‘two portals’; *n* = 14 256), a set that penetrated only the larger portal 1 (‘one portal’; *n* = 13 068), a set oriented so that the ∊-ionone ring with two chiral centers was closest to portal 1 (*n* = 13 662) and a set oriented in the opposite direction with this ∊ ring close to portal 2 (*n* = 13 662). The distribution of clashscores indicates a strong tendency for high-scoring models to belong to the ‘one portal’ set (Fig. 5 ▸) and oriented so that the ∊ ring is buried close to portal 2 (Fig. 6 ▸). Indeed, the absolute winner of the docking experiment belonged to both of these two sets and was seen with its β-ionone ring extending past portal 1 to nestle into a binding surface defined by the Ω1 loop with apparent steric and hydrogen-bonding complementarity (Fig. 7 ▸). To test the source of the observed orientation preference, we repeated the analysis with residues belonging to Ω1 and Arg351 in their alternate conformations and found that the number of clashes increased (on average +3 for Ω1-b and +1 for Arg 351-b), yet the strong (*p* < 0.00001) orientation preference was retained. Deleting residues belonging to Ω1 and truncating Arg351 to alanine shifted the frequency distributions to fewer clashes (−8 on deleting Ω1; −3 on truncating Arg351) and preserved the approximately threefold orientation preference favoring burial of the ∊ ring close to portal 2.

## 4. Discussion   

### 4.1. Co-crystallization of carotenoid-binding proteins   

A molecular understanding of how photoprotective carotenoids are recruited to the human retina will potentially help with the prevention and treatment of AMD. The carotenoid-binding proteins GSTP1 and StARD3 bind zeaxanthin and lutein, respectively, with affinity and specificity (Bhosale *et al.*, 2004 ▸; Li *et al.*, 2011 ▸). Efforts to co-crystallize the xanthophyll–protein complexes are under way; however, it has been difficult to find conditions under which the hydrophobic ligands are soluble and also compatible with the crystalline protein state. The most promising route to an experimentally determined co-crystal structure may emerge by combining powdered lutein and preformed crystals of StARD3_LBD_ in aqueous solutions containing trace amounts of detergent. The crystals appear to acquire a golden-brown color over several weeks (Fig. 1 ▸ *b*) yet retain X-ray diffraction performance.

### 4.2. Structure of StARD3_LBD_ and model of its complex with lutein   

While these soaking experiments were in progress, we measured X-ray diffraction for crystals of apo StARD3_LBD_ and generated models of the lutein–StARD3 complex by rigid-body docking. StARD3 crystals diffracted X-rays to relatively high resolution limits at a synchrotron source, allowing the detection of alternative conformations for a residue of interest found inside the central cavity of this helix-grip folded protein and for the Ω1 loop close to the entrance to the cavity. The winner of the rigid-body docking analysis incurs 14 steric overlaps with atoms of the protein and potentially makes two hydrogen bonds. By comparison, experimentally determined structures of lutein in complex with chlorophyll-binding proteins experience at most two steric clashes and make between three and five hydrogen bonds. The docking procedure applied here should be viewed as a coarse-grid search and caution is warranted in making detailed inferences from the modeling outcome.

#### 4.2.1. A portion of lutein must extend beyond the tunnel-like cavity   

The lutein-docking experiment is nevertheless illuminating in several respects. Comparison of the molecular size of lutein with that of the cavity leads to the inescapable conclusion that at least a portion of the lutein molecule must bind outside the cavity. Indeed, surfaces just outside the cavity appear to be complementary to the shape and hydrogen-bonding capacity of its β-ionone ring (Fig. 7 ▸). This predicted mode of binding is unprecedented for START proteins and other helix-grip folded proteins. In all structures where a ligand complex is available, the ligand is completely ensconced within the central cavity (Thorsell *et al.*, 2011 ▸; Létourneau *et al.*, 2015 ▸). Leaving a portion of the molecule on the outside of the protein as proposed here has important implications for the molecular interactions necessary for the transport and metabolism of lutein.

#### 4.2.2. Orientation preference and implications for ligand recognition   

An unexpected outcome of the docking experiment was that the ∊-ionone ring, with two chiral centers, appears to fit better inside the cavity, with fewer clashes and additional potential hydrogen bonds associated with this orientation, on average, in the ensemble of docking trials (see Fig. 6 ▸). This pattern suggests an interesting model for ligand selectivity: the binding-pocket asymmetry apparent for the lutein–StARD3 model may positively identify lutein, which is inherently asymmetrical with β-ionone and ∊-ionone rings, and exclude the closely related xanthophylls zeaxanthin and *meso*-zeaxanthin, which exhibit symmetry and have two β-ionone rings. Consistent with this idea that symmetry plays a role in xanthophyll selectivity, GSTP1, which is homodimeric, is the zeaxanthin- and *meso*-zeaxanthin-binding protein in the human retina (Bhosale *et al.*, 2004 ▸). The winner of the lutein–StARD3 docking experiment makes several close contacts with the Ω1 loop (Fig. 7 ▸) and the side chain of Arg351, suggesting that these contribute to the molecular-recognition properties of StARD3. A strong orientation preference was retained in the docking experiment with Ω1 and the side chain of Arg351 adopting their alternate conformations or even when they were removed. These results lead us to believe that molecular inter­actions involving surfaces throughout and outside the tunnel-like cavity, including Ω1 and Arg351, probably act cooperatively to select a particular binding orientation and positively identify lutein.

#### 4.2.3. Second portal of StARD3   

Although two portals potentially allow solvent to access the cavity at opposite ends of StARD3, one of these is much larger. The minimum number of clashes encountered for lutein molecules docked so as to penetrate both entrances was 23, which is nine more than that observed for the best-scoring model docked so as to penetrate just the larger portal. We therefore predict that lutein binds deep within the cavity, that one of the ionone rings protrudes through the larger opening which is guarded by the Ω1 loop, and that there is a close approach to but no breach of the smaller opening. It will be interesting to compare these predictions with an experimental structure of the co-complex and/or more refined modeling experiments that apply energy-relaxation methods (Das & Baker, 2008 ▸; Leaver-Fay *et al.*, 2011 ▸).

#### 4.2.4. Allosteric trigger point   

StARD3_LBD_ belongs to the helix-grip protein family, and protein members with structural homology include START proteins and allergen proteins, as well as several biosynthetic enzymes. Inspection of these structures reveals that StARD3 and StARD1 differ from the others in sharing identical or highly conserved residues at three positions that appear to be important for ligand binding: Arg at position 351 (numbered as in StARD3; 188 in StARD1), an acidic residue at position 332 (Asp in StARD3; Glu169 in StARD1) and Gln at position 421 (position 258 in StARD1). In other START-family members these three residues are variable, and all three are not found together. The lutein-docking results suggest that Gln421 may be positioned so as to make a hydrogen bond to a hydroxyl group of lutein outside portal 1 (Fig. 7 ▸). Electron-density maps, now calculated to 1.74 Å resolution, show that Arg351 adopts two alternative conformations (Fig. 3 ▸ *b*), one of which forms a salt bridge to Asp332. Interestingly, this salt-bridged conformation is probably incompatible with lutein occupancy owing to steric overlap. Based on these observations, we suggest that the salt bridge found in cavities of StARD3 and StARD1 may act as an allosteric trigger point to communicate ligand binding to other components of the steroid-generating apparatus in the case of StARD1, and to retinal proteins and enzymes involved with xanthophyll transport and metabolism in the case of StARD3.

## 5. Conclusion   

The structure of the lutein-binding domain of StARD3 has been determined to 1.74 Å resolution, revealing alternative conformations for elements of protein structure that appear to be critical for ligand binding. Modeling experiments indicate that the biologically relevant ligand must protrude from the cavity entrance because it is not possible to completely fit a 30 Å long molecule of lutein into a cavity that measures only 20 Å. Asymmetry in the tunnel-like cavity may play a role in selecting lutein with its β- and ∊-ionone rings and discriminating against the other ocular carotenoids, zeaxanthin and *meso*-zeaxanthin, which each have two β-ionone rings.

## Supplementary Material

PDB reference: cholesterol and lutein-binding domain of human STARD3, 5i9j

## Figures and Tables

**Table 1:** Data collection and processing Values in parentheses are for the outer shell.

Diffraction source	Beamline 12.3.1, ALS
Wavelength (Å)	1.11583
Temperature (K)	100
Detector	ADSC Quantum 315r
Rotation range per image (°)	0.5
Exposure time per image (s)	0.2 and 1.5
Space group	*P*3_1_21
*a*, *b*, *c* (Å)	83.39, 83.39, 82.19
α, β, γ (°)	90, 90, 120
Mosaicity (°)	0.14–0.21
Resolution range (Å)	41.7–1.74 (1.79–1.74)
Total No. of reflections	301752 (16789)
No. of unique reflections	34160 (2466)
Completeness (%)	99.5 (98.6)
Multiplicity	8.8 (6.8)
〈*I*/σ(*I*)〉	25.8 (2.4)
*R*_r.i.m._	0.048 (0.879)
Overall *B* factor from Wilson plot (Å^2^)	32.6

**Table 2:** Structure solution and refinement Values in parentheses are for the outer shell.

Resolution range (Å)	37.2–1.74 (1.79–1.74)
Completeness (%)	99.5 (98.6)
σ Cutoff	−3
No. of reflections, working set	32447 (2624)
No. of reflections, test set	1712 (141)
Final *R*_cryst_	0.170 (0.255)
Final *R*_free_	0.192 (0.320)
Coordinate error† (Å)	0.18
No. of non-H atoms	
Protein	1799
Ligand and ions	27
Water	224
Total	2050
R.m.s. deviations	
Bonds (Å)	0.013
Angles (°)	1.16
Average *B* factors (Å^2^)	
Protein	29.5
Ligand and ions	43.3
Water	36.7
Ramachandran plot	
Most favored (%)	97
Allowed (%)	3
*MolProbity* score‡	1.4
Clashscore	4.4
	

† Maximum-likelihood-based. See equation (19) in Lunin *et al.* (2002 ▸).

‡ Chen *et al.* (2010 ▸).
